# Improving care for Lynch syndrome patients: integrating surveillance into England’s national bowel cancer screening programme

**DOI:** 10.1007/s10689-026-00539-2

**Published:** 2026-02-26

**Authors:** Kevin J. Monahan, Stephanie X. Poo, Fiona Lalloo

**Affiliations:** 1 St Mark’s Hospital Centre for Familial Intestinal Cancer, London, HA1 3UJ UK; 2 Department of Surgery and Cancer, Imperial College London, London, UK; 3 Manchester Centre for Genomic Medicine, Manchester University Hospitals NHS Foundation Trust, Manchester, UK

**Keywords:** Lynch syndrome, HNPCC, Hereditary nonpolyposis colorectal cancer, Colonoscopy

## Abstract

Quality assurance, timeliness and equity of access to colonoscopy for people with Lynch syndrome (LS) in England has historically been highly variable. The LS-Bowel Cancer Screening Programme (LS-BCSP) launched in July 2023, delivers high quality colonoscopy to the average-risk population, from colonoscopists who have undergone high-level accreditation, utilising the existing average-risk BCSP infrastructure. Eligible individuals have a genetic diagnosis of LS. Comprehensive retrospective diagnoses of LS in England (since the 1990s) were ascertained from 17 regional genetics services. The National Disease Registration Service (NDRS) developed a registry of eligible individuals, and a portal for prospectively diagnosed cases. An existing national screening IT framework was adapted to incorporate disease-specific clinical pathway information, linked to existing national guidelines. Nationally standardised training for BCSP teams was delivered to > 2000 staff from 64 national screening centres in 2023. By November 2024, 10,913 eligible individuals were identified, with 150–250 new diagnoses added each month. A historical backlog of > 1000 patients overdue colonoscopy surveillance was cleared by January 2024. Diagnostic outcomes of LS patients from the first two years of LS-BCSP will be available to facilitate evaluation of the successes and failures of the LS_BCSP. This evaluation will include diagnostic outcomes, stratified by demographic and socioeconomic status, genotype, measures of colonoscopy quality and regional variation. This novel programme includes complete ascertainment of the national LS population in England, without requirement for referral. Individuals with LS now have access to high-quality, timely colonoscopy through an accredited programme which is quality-assured along the entire pathway.

## Introduction

Lynch syndrome (LS) is the most prevalent autosomal dominantly inherited cancer predisposition disorder, caused by pathogenic variants in DNA mismatch repair (MMR) genes (*MLH1*, *MSH2*, *MSH6*, and *PMS2*) as well as deletions in *EPCAM* that induce epigenetic silencing of *MSH2* [1–4]. This results in an increased lifetime risk of colorectal (CRC), endometrial and a spectrum of other epithelial malignancies [5, 6]. As such, LS carriers are enrolled into cancer prevention programmes including gene-specific colonoscopic surveillance, aspirin chemoprevention and risk-reducing gynaecological interventions [5, 7, 8].

Given the elevated cumulative CRC risks in LS, the British Society of Gastroenterology (BSG) guidelines for the UK recommend two-yearly colonoscopic surveillance starting from ages 25 or 35, depending on the affected gene [7]. This recommendation is grounded in evidence from longitudinal cohort studies and registry data consistently demonstrating reductions in CRC incidence and mortality in LS patients undergoing colonoscopic surveillance [9, 10]. Therefore, people with LS may expect to have 20–30 standard of care colonoscopies over their lifetime, and poor quality colonoscopic pathways may result in avoidable CRC diagnoses [11]. As such their experience of bowel preparation, the quality of the colonoscopy itself, including comfort, recovery, follow-up and recall create a specific challenge for clinical services which is not reflected in other patient populations (other than those who also require lifelong colonoscopic surveillance). Patients can find the experience of undergoing colonoscopy burdensome, may have a difficult episode which reduces their desire to have further procedures, or be ‘lost to follow-up’ either due to heterogeneous recall in secondary care, or difficulty accessing colonoscopy from primary care [12].

The protective effects of surveillance are contingent upon high quality colonoscopy, which reflects how effectively the colonic mucosa is examined. Several factors such as adenoma detection rate, caecal intubation rate, bowel preparation quality, and withdrawal time are established determinants of CRC prevention, early detection and deaths in the average-risk population [13, 14]. These quality indicators are equally relevant in LS, where high-quality complete colonoscopies performed at less-than-three-year intervals have been associated with a reduction in interval CRC rates [15]. While it is still unclear whether post-colonoscopy CRC rates in LS are driven primarily by poor colonoscopy quality or underlying biological factors, optimising colonoscopy quality is essential to maximise the full preventative potential of surveillance.

The responsibility of organising colonoscopic surveillance and follow-up appointments has been variable and heterogeneous, with limited recall and a lack of standardised quality control. Once people are diagnosed with LS by clinical genetics services, there has been a reliance on local endoscopy units to have local systems to schedule patient appointments. However, despite national guidelines, significant variability remains in clinical care, quality and timeliness of testing and surveillance recall for individuals with LS. This was corroborated by a national survey on hereditary CRC services which highlighted clinicians’ poor awareness of and adherence to guidelines, with some perceiving that responsibility for patient care lies with someone else [16, 17].

In 2017 national guidelines in the UK recommended universal testing for LS in people diagnosed with CRC. However, it was recognised that significant gaps in care needed to be addressed alongside diagnostic testing. A multi-society meeting at the Royal College of Surgeons (RCS) was organised by the patient charity Bowel Cancer UK, and included patient representatives, the BSG, Association of Coloproctology of Great Britain and Ireland (ACPGBI), Bowel Disease Research Foundation (BDRF), and National Cancer Research Institute (NCRI). They made three recommendations:

1. A dedicated clinical champion for hereditary CRC within every colorectal MDT to deliver diagnostic testing and coordinated care.
2. The development of a National LS registry.
3. A quality assured surveillance programme for LS patients​

**Fig. 1:**
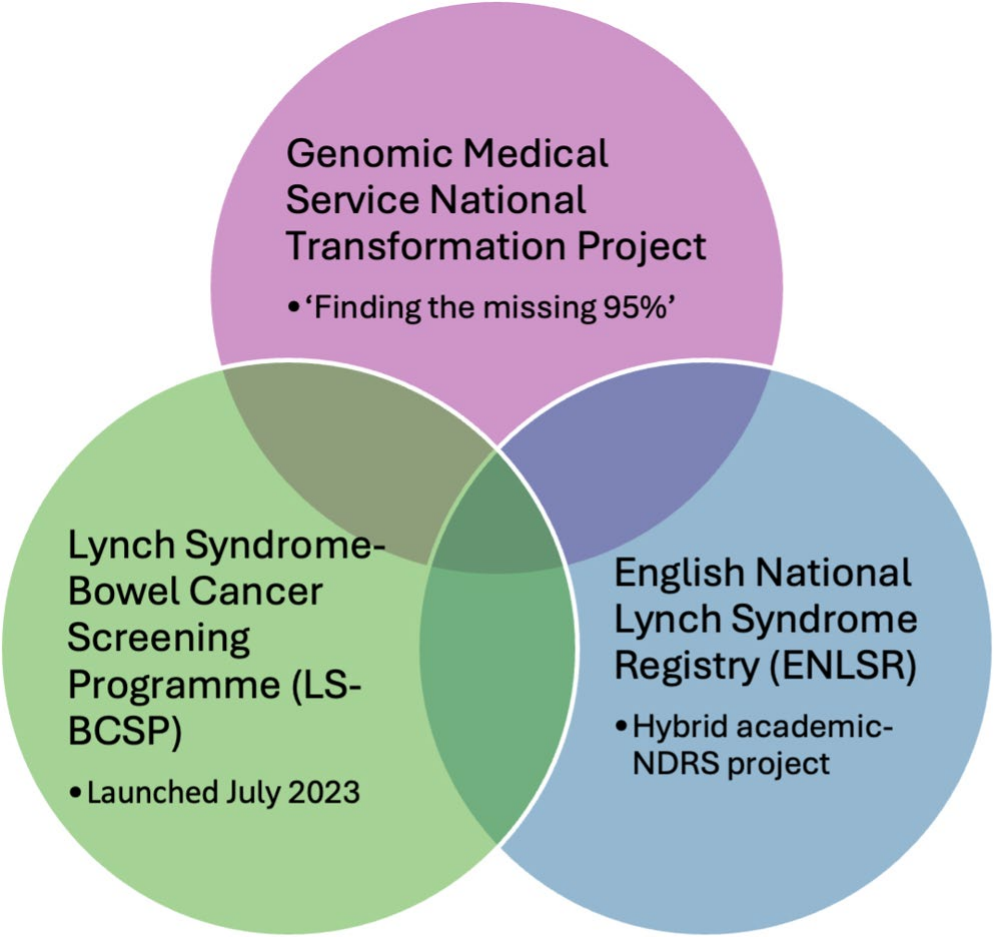
The English National Lynch Syndrome Projects [19]. Abbreviations: NDRS, National Disease Registration Service

**Fig. 2:**
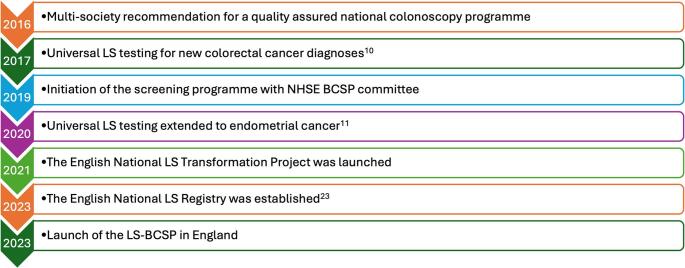
Key milestones in UK LS identification and management. Abbreviations: BCSP, Bowel Cancer Screening Programme; LS, Lynch syndrome; NHSE, National Health Service England

In order to develop a systematic approach for the diagnosis and management of LS in England, three overlapping projects were eventually launched which mirrored the agreed priorities outlined above (Figs. 1 and 2) [18, 19]:

By streamlining genetic testing and referral pathways, the English National Transformation Project is expected to increase identification of LS carriers who may otherwise remain undiagnosed and through this registry, these patients may be automatically linked into screening programmes, forming the foundation for the LS-BCSP.

The Transformation Project ensured that all patients diagnosed with LS since genetic diagnosis commenced in the 1990s were identified via all diagnostic services in England. A national portal developed by National Disease Registration Service (NDRS) allowed prospective diagnoses to be entered.

The ENLSR, coordinated by the NDRS, was established as a centralised national database of LS carriers, designed to address fragmentation of care and ensure fair access to preventative interventions such as colonoscopic surveillance [20]. It links cancer records with genomic data, collates historical diagnoses (from 1995 to 2023) entered retrospectively by clinical genetics services and captures new diagnoses prospectively through a national portal, thereby providing a comprehensive national resource with complete population ascertainment. Data were securely shared with NDRS under the legal framework of Sect. 254 of the Health and Social Care Act 2012. This infrastructure provided the foundation for a robust, centralised LS registry that supports population-level surveillance, targeted screening, and future service planning.

Therefore, those previously and newly diagnosed with LS would have access to optimal colonoscopic follow-up, with equity to access and complete population ascertainment. Between 2020 and 2024, the period of the National Transformation Project, we observed a 255% improvement (from a total of 545 in 2020 to a total of 1394 in 2024) in the diagnosis of LS and identified 10,913 eligible patients (both retrospective and prospectively diagnosed cases) from the ENLSR designed to deliver patients to a new national bowel cancer screening programme.

## The LS-Bowel cancer screening programme (LS-BCSP)

Following the Independent Review of Adult Screening Programmes by Professor Sir Mike Richards commissioned by NHS England (NHSE) in 2019, there was a movement to expand and prioritise targeted screening pathways for high-risk populations, including LS [21]. Discussions between clinical experts, Bowel Cancer UK and senior NHS decision makers led to an options appraisal about delivery of colonoscopic surveillance integrated within, or remaining outside the existing BCSP infrastructure [19].

The benefits of the LS-BCSP must however be balanced against the increased demand on BCSP services. Modelling data developed for this programme estimated an additional 700–1500 index LS diagnoses per year and incorporated increased rates of LS diagnosis related to the National Transformation Project. This translated to an estimated 5,000 further colonoscopies in the first year which is projected to increase to 14,000 procedures by 2028; with this estimate is expected to rise year-on-year as case ascertainment increases [22]. This initial increase in demand will be driven in-part by the need to provide surveillance to the existing LS population and address the backlog of patients overdue a colonoscopy prior to programme entry. As LS colonoscopic surveillance becomes fully integrated within the BCSP framework, annual capacity was expected to stabilise with future increases driven largely by incident cases. Workforce modelling and feasibility assessments indicate that with targeted planning and resource allocation, these additional demands can be accommodated within the BCSP infrastructure, however ongoing evaluation is necessary to ensure that the increased workload can be effectively managed.

## Structure of the LS-BCSP

The NHS BCSP is structured into five regional screening hubs that oversee 64 JAG-accredited screening centres, each linked to the nearest General Practitioner (GP) to provide an equitable spread of services across England [23]. This regional structure forms the backbone for the delivery of LS-colonoscopic surveillance within BCSP.

Each regional genetics service, along with the LS service at St. Mark’s Hospital, submit data about individuals with a confirmed genetic diagnosis of LS to the ENLSR via an electronic portal developed by NDRS. A minimum dataset was developed, including essential identifiers (first name, surname, date of birth, NHS number, gender, and postcode), date of LS diagnosis, and LS gene. NDRS maintains the ENLSR and transfers information to the Bowel Cancer Screening System (BCSS) for invitation to the screening programme. The BCSS IT system maintains a prospectively collected database detailing every stage of the pathway from invitation to follow-up, and in addition, has an inbuilt mechanism for ensuring automated invitations and recalls [23]. Eligible individuals are invited from the age of 25 for *MLH1*, *MSH2* and *EPCAM* carriers, and 35 for *MSH6* and *PMS2* carriers in line with national guidelines, or from the time they are entered into the LS registry if diagnosed above these ages and are due for colonoscopy [7]. Individuals at-risk of LS but without a confirmed MMR pathogenic variant are managed outside the LS-BCSP.

A BCSP colonoscopist must meet strict competency standards monitored by the Joint Advisory Group on Gastrointestinal Endoscopy (JAG). This includes undertaking a BCSP accreditation entry examination after they have completed a minimum of 1,000 lifetime procedures, where they must pass an online multiple-choice assessment and complete two directly observed colonoscopies evaluated by independent expert endoscopists [23, 24]. High-quality colonoscopy performance is further maintained through continuous monitoring of key performance indicators (such as adenoma detection rate, polyp detection rate, caecal intubation rate, withdrawal time, patient comfort and complication rate) alongside regular audits and feedback mechanisms that evaluate adherence to programme standards, clinical outcomes and patient experience [25].

Patient experience is particularly important in LS surveillance, as LS carriers often require multiple colonoscopies throughout their lifetime. To support this, BCSP offers personalised care through Specialist Screening Practitioner (SSP) appointments and quality-assured processes designed to optimise procedural outcomes. SSPs are employed within BCSP and are embedded within BCSP-accredited screening centres where colonoscopies are delivered while working in close coordination with endoscopy staff. Furthermore, online e-learning modules, clinical resources and dedicated training workshops were developed and delivered to healthcare professionals working in BCSP and clinical genetics services to equip them with knowledge of testing pathways and LS-BCSP implementation. Collectively, these safeguards ensure that LS patients consistently receive high-quality colonoscopy delivered equitably across the nation.

The pathway begins with a pre-invitation letter, followed by an appointment with a SSP and colonoscopy at their local screening centre (Fig. 1). Individuals who have had a colonoscopy within the previous two years will have their surveillance intervals reset, and a new appointment is scheduled. Patients who do not attend their appointments are managed through repeated LS-BCSP invitations and failure to attend despite this will result in automatic recall after 2 years, with primary care physicians copied into correspondence at each stage to encourage general practitioner awareness. Furthermore, patients who have recently been diagnosed with CRC will be offered a colonoscopy follow-up at 12 months under symptomatic services (typically with their usual hospital clinician or cancer teams), and if this is satisfactory, subsequent colonoscopies will be scheduled every two years under the LS-BCSP.

During the SSP appointment, patients undergo a thorough health and fitness assessment to ensure suitability for colonoscopy, and partake in discussions about sedation, dietary instructions and bowel preparation. If deemed fit and suitable, a colonoscopy is scheduled in the proceeding weeks. Two main outcomes are possible following colonoscopy: (1) Cancer is diagnosed prompting staging investigations and cancer multidisciplinary team management, or (2) For all other results, they will be recalled for a colonoscopy every 2 years until the age of 74 in line with existing BSG guidelines, with an option to extend surveillance beyond this age [7]. For individuals in whom biopsies or polypectomies are performed, results are discussed as part of a structured follow-up process whereby SSPs explain the histology results within a week of the colonoscopy, discuss the outcome of this colonoscopy and outline subsequent management. The SSPs act as the key point of contact for patients within the BCSP framework supporting patients throughout the entire pathway, and are responsible for patient preparation, communication of results, offering support and coordinating follow-ups, in both the LS and average-risk BCSP populations.

**Fig. 3:**
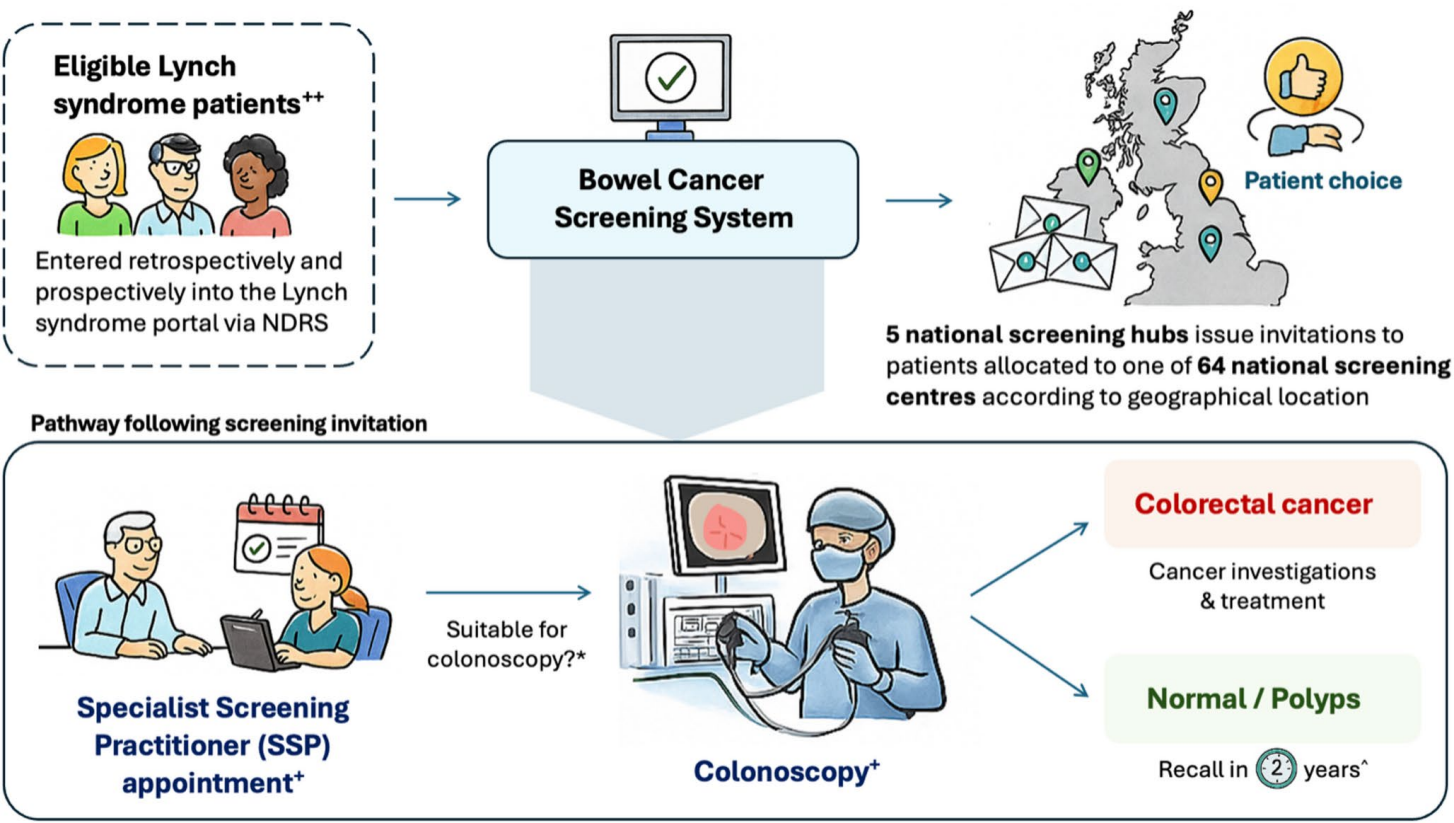
Lynch syndrome-Bowel Cancer Screening Programme (LS-BCSP) pathway. Abbreviations: BSG, British Society of Gastroenterology; BCSP, Bowel Cancer Screening Programme; FIT, Faecal Immunochemical Test; LS, Lynch syndrome; NDRS, National Disease Registration Service; SSP, Specialist Screening Practitioner. Footnotes: ++Eligible Lynch syndrome patients include adults with a confirmed pathogenic variant in *MLH1*, *MSH2*, *EPCAM*, *MSH6* or *PMS2*. Biennial surveillance colonoscopy begins at the age of 25 for *MLH1*/*MSH2*/*EPCAM* carriers and the age of 35 for *MSH6*/*PMS2* in line with the BSG guidelines [7]. +Repeated non-attendance to appointments will result in automatic recall in two years. *During SSP appointments, LS patients will undergo a health and fitness assessment to confirm ongoing suitability for colonoscopy. ^Patients will be invited for colonoscopy surveillance every 2 years until the age of 74, after which they may opt in for continued surveillance pending health and fitness assessments.

Throughout this process, patients are offered a choice, from selecting a different screening centre to the one allocated to them, deferring or bringing forward appointments due to cancer treatments, planned surgery or pregnancy, and opting out of BCSP in favour of symptomatic follow-up. Patients may also choose to withdraw from LS-BCSP but remain on FIT-based surveillance, or be removed from both LS- and FIT-based BCSP. Since its inception, over 10,000 LS carriers have been invited for colonoscopic surveillance under LS-BCSP, with more than 5,000 diagnostic tests completed. This represents a major milestone in the delivery of population-level surveillance for LS patients in England, upheld by a robust national infrastructure with real-time data collection, continuous performance monitoring and quality assurance (Fig. 3).

## Conclusions and future directions

Through coordinated multifaceted initiatives of the English National LS Transformation Project and NHSE, significant progress has been achieved in LS testing, surveillance and management. A historical backlog of > 1000 patients overdue colonoscopy surveillance was cleared by January 2024. According to the 2024 ‘Finding the missing 95%: Unlocking the potential of Lynch syndrome services’ report by Bowel Cancer UK, marked improvements have occurred since the 2018 FOI request, with majority of hospitals now testing tumours for MMR deficiency using IHC or MSI resulting in an increased number of LS diagnoses made nationally [26]. However, variation in care persists between the UK nations, for example the LS-BCSP is not yet available in Wales, Scotland or Northern Ireland, a drawback of the UK devolved healthcare systems.

Comparable national screening programmes for high-risk populations can help inform benchmarks for implementing and optimising LS management. A centralised registry design was adopted by the ENLSR; however, further work is still required to risk stratify and personalise surveillance according to genotype and patient-specific factors in LS. Nonetheless, such a registry could be adapted to other high-risk cancer populations requiring structured surveillance. Indeed, the National Inherited Cancer Predisposition Register (NICPR) was established for this purpose in 2023, adapting the ‘exemplar’ ENLSR for a range of other Mendelian syndromes, amalgamating individuals carrying cancer susceptibility genes into a unified national dataset [27].

All national BCSP centres in England were expected to participate in the new LS_BCSP, and over 2,000 BCSP staff participated in one of 8 half-day of training sessions about the processes within the new LS-BCSP, and clinical issues related to management of LS. Furthermore, standardised resources (such as Aspirin information leaflet) as well as information and e-learning modules for primary care physicians have been developed to facilitate consistent dissemination of information [28]. However, the LS-BCSP was designed to specifically deliver systematic quality assured colonoscopy, and not other aspects of their holistic care. The responsibility for non-colorectal risk management and chemoprevention counselling remains under the remit of clinical genetics services or other highly specialist services. Where such issues are raised, SSPs may direct individuals back to specialist services or relevant pathways. Nevertheless, although not mandated through the LS-BCSP, the new programme provides opportunities for other interventions, such as the use of aspirin, or referral for gynaecological risk management, with LS-BCSP staff increasingly trained to address common patient queries and facilitate signposting to appropriate resources [29].

Evaluation of the success and failures of the LS-BCSP are focused on clinical outcomes and key performance indicators, which will form an evidence base for policy development, gene-specific surveillance and ongoing improvements to the programme. Data from the first unselected cohort of LS patients will provide valuable real-world insights on programme outcomes, including surveillance uptake, colonoscopy quality and neoplasia detection rates within a national screening framework. Recent UK LS colonoscopy outcomes demonstrated high adenoma and advanced adenoma detection rates of 31.3% and 6.8% respectively achieved under high quality surveillance, reflecting outcomes achievable within a quality-assured BCSP model and provides a benchmark for neoplasia detection and performance standards for the LS-BCSP [30]. Patient feedback will also be integral to shaping service delivery and ensuring that the service aligns with their needs. Building on the successes of the LS-BCSP in England, extending this level of equity to the devolved UK nations will be an important future priority to achieve nationwide parity in high-quality surveillance.

In England, the new LS-BCSP demonstrates the feasibility of implementing a structured national surveillance programme for individuals with LS and provides a framework by which international healthcare systems may adapt and deliver LS surveillance in devolved UK nations and globally.

## Data Availability

No datasets were generated or analysed during the current study.
